# Primary and Delayed Primary Suture for Fractures

**Published:** 1918-08

**Authors:** 


***Primary and Delayed Primary Suture in the Treatment of War Fractures.*** By William S. BAER, Major, M. R. C., assistant director, Orthopedic Surgery, A. E. F. *Journal of the American Medical Association*, May 25, 1918, ol. LXX, No. 21, p. 1531.

The treatment of war fractures is the treatment of wounds of the soft parts plus the added difficulties which the injuries to bony tissue involves. The principles to remember are :

1. All battle wounds are to be considered as infected.

2. It is necessary to remove all projectiles, clothing, and devitalized tissue as early as possible; at least before the twelfth hour after injury.

3. These wounds can then be considered as aseptic in character and a primary suture made, thus converting compound fractures into simple fractures, and appropriate treatment for these simple fractures thereupon instituted.

Experience has shown, says Baer, that from 80 to 95 o/o of cases may be treated by means of primary suture.

I. *Technique of Primary Suture* : Absolute asepsis throughout. Skin disinfected with iodin or other method. The skin edge with the tract made by the projectile is removed. All projectiles and all devitalized tissue are removed. Complete hemostasis. Approximate skin edges without tension.

It has been said that 10 o/o of battle wounds cannot be primarily closed, even if received within twelve hours after injury. Such cases are (1) those in which shock is so great that a complete operation cannot be done ; (2) those in which for any reason whatever all foreign matter cannot be removed; (3) those in which the loss of substance is so great that complete closure is impossible.

Cultures must be taken at time of suture and some wounds must be reopened immediately on bacteriological report, e.g., those primarily infected with the virulent types of streptococci.

II. *Delayed Primary Suture* : Performed from three to ten days after the primary operation, where (usually for military reasons) a primary suture has been deemed inadvisable. The primary operation is performed as in primary suture, the only difference being that the skin edges are not brought together until later. Primary suture should never be done where prompt evacuation is necessary or where a roentgenologist and a bacteriologist are not present. Good team work of surgeon, roentgenologist, and bacteriologist is absolutely necessary. Patients with primary suture should be under observation for at least ten days; so that the wound may be watched and not subjected to the traumatism of transportation. Where this cannot be done delayed primary suture is practised.

III. *Fractures*: Baer insists that by the use of the principle of primary and delayed primary suture the entire subject of the treatment of fractures has been changed. One no longer treats compound fractures except by the simple procedure followed in the case of a simple fracture. But such cases should be operated upon within eight hours of the casualty and the handling of the wounded must be accomplished with this end in view. Hospitals adequately staffed and of sufficient size must be located in the zone of the advance so that the wounded may be handled in good time and be permitted to rest at the hospital in which they have been operated for at least ten days.

